# Enzyme-Assisted Nucleic Acid Amplification in Molecular Diagnosis: A Review

**DOI:** 10.3390/bios13020160

**Published:** 2023-01-19

**Authors:** Meiling Wang, Hongna Liu, Jie Ren, Yunqi Huang, Yan Deng, Yuan Liu, Zhu Chen, Franklin Wang-Ngai Chow, Polly Hang-Mei Leung, Song Li

**Affiliations:** 1 Hunan Key Laboratory of Biomedical Nanomaterials and Devices, Hunan University of Technology, Zhuzhou 412007, China; 2 Hengyang Medical School, University of South China, Hengyang 421001, China; 3 Department of Health Technology and Informatics, The Hong Kong Polytechnic University, Hong Kong 999077, China

**Keywords:** nucleic acid amplification technology, enzymes, polymerase chain reaction, isothermal amplification, CRISPR-Cas, clinical application

## Abstract

Infectious diseases and tumors have become the biggest medical challenges in the 21st century. They are driven by multiple factors such as population growth, aging, climate change, genetic predispositions and more. Nucleic acid amplification technologies (NAATs) are used for rapid and accurate diagnostic testing, providing critical information in order to facilitate better follow-up treatment and prognosis. NAATs are widely used due their high sensitivity, specificity, rapid amplification and detection. It should be noted that different NAATs can be selected according to different environments and research fields; for example, isothermal amplification with a simple operation can be preferred in developing countries or resource-poor areas. In the field of translational medicine, CRISPR has shown great prospects. The core component of NAAT lies in the activity of different enzymes. As the most critical material of nucleic acid amplification, the key role of the enzyme is self-evident, playing the upmost important role in molecular diagnosis. In this review, several common enzymes used in NAATs are compared and described in detail. Furthermore, we summarize both the advances and common issues of NAATs in clinical application.

## 1. Introduction

Nucleic acid is an essential substance for life. Deoxyribonucleic acid (DNA) carries genetic information and is responsible for encoding amino acid sequences. Ribonucleic acid (RNA) is responsible for the regulation of gene expression. Additionally, nucleic acids are used as important biomarkers for medical diagnostics [1]. Nucleic acid amplification technologies (NAATs) have been proved to be the most effective way to detect these biomarkers. As the developments of NAATs are increasingly updated, they have been incorporated into every field of medical research, even our daily life [2,3,4]. In 2016, the World Health Organization (WHO) found that 3 of the top 10 causes of death globally were related to infectious diseases, with lower respiratory infections remaining the most deadly communicable diseases, killing 3 million people worldwide in 2016 [5]. With early detection and treatment, the global death toll can be reduced. NAATs play a significant role in early detection. For example, during 2010–2017, HIV-related death rates decreased by 48.4% (from 9.1 to 4.7) in America, and the rates of death decreased by 36.6% overall in 2010–2018. This is thought to be the result of early diagnosis and prompt treatment and remains necessary for continuing reductions in HIV-related deaths [6]. The most critical component of these technologies is enzymes. It is with the action of enzymes that NAAT can continue to be developed and utilized in the medical field for early disease detection.

NAATs primarily consist of polymerase chain reaction (PCR) and isothermal amplification. Mullis proposed PCR in 1983, which has become the gold standard of the molecular diagnostics industry [7]. This technique has become the most valuable in molecular biology, diagnostics and other fields [8,9]. Due to the professional operation and thermal cycling instruments that PCR requires, it is not suitable for clinical diagnosis in areas with limited resources or outside laboratories. Isothermal amplification technology (IAT) is a nucleic acid amplification technology comparable to PCR. It does not rely on the change in temperature, nor does it need expensive instruments, such as thermal cyclers, to amplify nucleic acids [10,11,12]. More recently, clustered regularly interspaced short palindromic repeats (CRISPR) have become the research focus of molecular diagnosis due to their convenience, rapidness and high accuracy. They have been used in combination with a variety of NAATs and have been successfully applied in the clinical market. Commonly used IAT methods include Loop-mediated isothermal amplification (LAMP), Recombinase polymerase amplification (RPA), Rolling circle amplification (RCA), Strand displacement amplification (SDA) and Nucleic acid sequence-based amplification (NASBA).

Enzymes are proteins or RNA with highly specific catalytic activity produced by living cells. The essence of life is to produce chemical reactions, and this is the most critical step of enzymes. In molecular diagnosis, the activity of enzymes can directly determine the efficiency of detection. In this study, we introduce the principle of PCR, five isothermal amplification technologies (LAMP, RPA, RCA, SDA, NASBA) and CRISPR, along with the core enzymes that are required for each.

## 2. Nucleic Acid Amplification Technologies

The emergence of PCR has had a huge impact on molecular biology and diagnostics, and this technique can quickly amplify a large number of copies with a small number of DNA templates [13]. The invention of PCR technology is a revolutionary initiative and a milestone in the biomedical field. Compared with traditional PCR, isothermal amplification does not require changes in temperature, is fast and has a high specificity, so it has become a research hotspot in molecular diagnosis. IAT is on par with PCR in terms of amplification efficiency, even surpassing it. A comparison of PCR and five different isothermal amplification techniques is provided in Table 1.

### 2.1. Polymerase Chain Reaction

#### 2.1.1. Enzymes

Taq polymerase is a thermostable DNA polymerase that is derived from Thermus aquaticus and is capable of growth at 70–75 °C; it is the most widely used PCR enzyme [25,26]. Taq has a high thermal stability and can withstand higher thermal denaturation steps of PCR. Studies have shown that this enzyme can be activated at 95 °C for a long period of time, even at 95 °C for half an hour; it still retains more than 50% activity [27]. Taq is one of the few DNA polymerases that contain a polymerization of 5′–3′, which is highly sensitive to temperature, so the reaction can stop amplification at lower temperatures [28]. This property is also an important part of the specific detection of PCR products. Holland et al. excised the radiolabeled DNA probe at the 5′ end of the Taq DNA polymerase by using the 5′–3′ exonucleation activity to realize the specific detection of the PCR product through the change in the radioactive signal [14]. However, it lacks 3′–5′ exonuclease activity and correction, which reduces its fidelity in PCR amplification. Of course, in addition to Taq polymerase, double-stranded DNA fragments are linked specifically by DNA ligase, with the final target of interest amplified exponentially, also known as ligase chain reaction (LCR), which is mainly used in the study of point mutation. The common Taq polymerase is used in conventional PCR but is prone to non-specific amplification caused by mismatches and produces primer-dimers. Hot-start enzymes are an ideal choice for higher-specificity amplification. After modification, it is usually heated at 95 °C for 5–10 min before the active center is exposed to produce polymerization, and it does not exert enzyme activity before 72 °C, so the template mismatch at low temperatures can be effectively avoided, and dimers and non-specific amplification can be avoided. Hot-start enzymes such as Relia^TM^ HS effectively prevent non-specific PCR products, thus allowing for the amplification of troublesome samples such as single copy gene amplification, multiplex PCR and ultra-long fragments.

#### 2.1.2. Reaction Principle and Improved Methods

**Figure 1:**
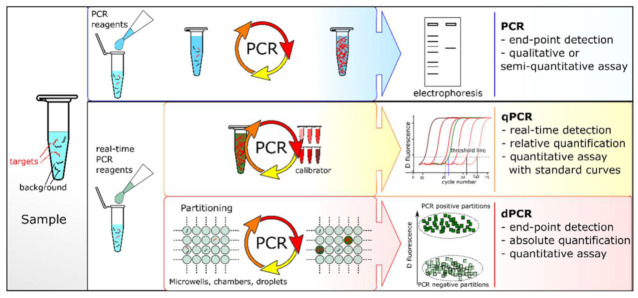
PCR comparisons: end-point PCR, qPCR and dPCR. Reprinted with permission from Ref. [33].Copyright 2018, Quan et al.

Under the catalysis of the DNA polymerase, PCR amplification technology takes the DNA parental strand as a template and specific primers as an extension starting point. The strand is replicated through denaturation, annealing and extension steps [29]. However, the specificity of traditional PCR is lower for fragments with a high GC content. Sarkar et al. proposed that formamide could eliminate most of the non-specific products of GC-rich segments, improving the amplification efficiency and specificity [30]. Until recently, there were three PCR systems: end-point PCR, qualitative or real-time PCR (qPCR) and digital PCR (dPCR) (Figure 1). The conventional PCR should be analyzed by gel electrophoresis at the end of the reaction and detected after fluorescence staining. Heid et al. reported adding fluorescent groups into the qPCR system, quantifying unknown templates according to the Cq value of the fluorescence curve [31]. dPCR, introduced in 1999, can directly count target molecules without relying on any external markers [32]. dPCR is based on a microfluidic chip and microdroplet method. qPCR and dPCR use the same amplification reagents and fluorescent labeling systems, but the key difference between them is the strategy of measuring the number of target sequences. In qPCR, quantification is based on the analysis of the fluorescence signal in the exponential phase, and the response is monitored throughout the process. In contrast, dPCR calculates the target concentration in reverse by collecting the fluorescence signal at the end of the reaction and using the number of total positive segments [33]. In addition, Hamberlain proposed Multiplex PCR (MPCR) for the prenatal and postnatal diagnosis of Duchenne muscular dystrophy [34]. This method can amplify multiple targets with a single PCR reaction, which is another important milestone in the development of PCR. At present, MPCR technology has been widely used in scientific research, disease diagnosis and other fields, including next-generation sequencing (NGS) of the whole genome database [35].

### 2.2. Loop-Mediated Isothermal Amplification

#### 2.2.1. Enzymes

In 2000, Notomi and colleagues invented Loop-mediated isothermal amplification (LAMP), and this became the gold standard for point-of-care (POC) amplification methods as soon as it was published. By using a special primer design method and chain replacement enzyme, a small number of target DNA can be amplified to millions in 60 min [16]. In the LAMP, the most critical component is the use of the Bst DNA Polymerase. It has natural reverse transcriptase activity and is a multifunctional enzyme with strong chain replacement activity. The Bst DNA Polymerase is derived from the thermophilic Bacillus species, which has an optimal reaction temperature of 60–65 °C. In addition, it has a 5′–3′ polymerase fragment but lacks the 5′–3′ exonuclease activity [36]. As a result, compared with other polymerases, the Bst DNA polymerase has stronger thermal stability, chain replacement activity and polymerase activity, so it is more suitable for isothermal amplification. It is also preferred for the synthesis of DNA chains with a high GC content for DNA sequencing [37,38]. It is worth noting that the use of the Bst DNA polymerase should not exceed 70 °C and cannot be used for thermal cycle sequencing or PCR. With the development of molecular biology technology, the Bst 2.0 DNA polymerase has been upgraded to the Bst 3.0 DNA polymerase. Compared with the wild Bst DNA polymerase, these improved enzymes can effectively improve the amplification speed, yield, salt tolerance and thermal stability [39,40].

#### 2.2.2. Reaction Principle and Improved Methods

**Figure 2:**
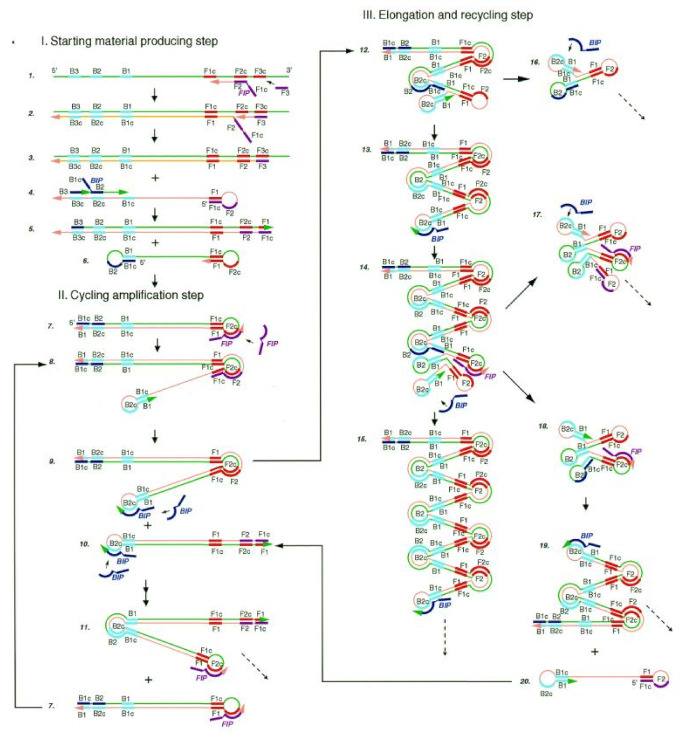
Steps in the LAMP mechanism. A pair of inner primers (BIP) with a folding structure and a pair of outer primers (FIP) for replacing single-strand products are required. Under the action of FIP, BIP and the Bst DNA polymerase with chain displacement activity, dumbbell intermediate products with a ring structure at both ends are generated. The amplification products of dsDNA concatemers were amplified by the double action of initiating extension in the ring region of the BIP recognition intermediate and self-initiating extension at the 3′ end of the folding structure. Reprinted with permission from Ref. [15]. Copyright 2018, Wong et al.

LAMP is widely used in the field of molecular diagnosis. In this method, four specific primers are designed for six regions of target genes. Under the action of the Bst DNA polymerase, the gene template, primers and chain replacement, DNA were amplified at a constant temperature of 60–65 °C (Figure 2). The final products can be detected by both real-time assays and end-point methods [41].

As mentioned above, LAMP alone is far from enough, so several LAMP-based nucleic acid amplification methods are extended. Reverse transcription-loop-mediated isothermal amplification (RT-LAMP) reaction can occur under the action of the Bst 3.0 DNA/RNA polymerase to specifically amplify RNA pathogens. Kashir has successfully used LAMP to detect COVID-19 [42]. Teoh et al. used nine primers with RT-LAMP to detect all four DENV serotypes and their different genotypes [43]. They used a primer combination for the highly conserved region of DENV 3′UTR to detect 11 different strains of DENV genotypes; the detection limitation was at least 100 copies.

The conventional LAMP can only detect nucleic acid qualitatively, not quantificationally. In order to solve this problem, digital LAMP (dLAMP) is proposed [44,45]. According to the equipment used in the compartmentalization process, the dLAMP technologies can be divided into three categories, including chamber-based microfluidic dLAMP, droplet microfluidic dLAMP and other types of dLAMP by which compartmentalization is achieved without utilizing microfluidics [46]. dLAMP can not only quantify absolute nucleic acid concentration but also inherits the advantages of LAMP technology, such as the low instrumentation complexity, high specificity and strong tolerance to inhibitors, and it even increases the detection limit [47]. However, LAMP requires four or six primers, so it has a high requirement on primers. Secondly, LAMP has a strong sensitivity and it is particularly easy for it to form aerosols, resulting in a false-positive influence on detection results. It is worth mentioning that the amplified products cannot be used for cloning sequencing but can only be used for judgment.

### 2.3. Recombinase Polymerase Amplification

#### 2.3.1. Enzymes

RPA was developed by Piepenburg et al. in 2006 [48]. Since it was first reported, RPA has been recognized as a unique technology and has been patented by TwistDx in the UK. It is a revolutionary innovation in the field of DNA diagnosis. The core of recombinase polymerase amplification (RPA) technology lies in the use of three enzymes: the recombinase (T4 UvsX), the ssDNA binding protein (SSB), named gp32, and the Bsu DNA polymerase. UvsX is the RecA/Rad51 ortholog [49]. RecA/Rad51 plays an important role in double-stranded DNA repair and homologous recombination. UvsX and other DNA-binding proteins or cofactors can form nucleic acid-protein complexes with single-stranded DNA. The complex hybridizes with the complementary region of the target DNA to further complete the strand replacement reaction [50]. In addition, UvsY is a recombinant regulatory protein of T4 phage. The UvsY recombination mediator protein is used as a recombinase loading factor to catalyze the T4 UvsX activities [51]. The molecular weight of the UvsY protein is 16 KDa, which can enhance the activity of the DNA-dependent ATPase of the UvsX protein, thus promoting chain replacement [48]. T4 gp32 is an SSB which is necessary for DNA replication and repair [52]. More importantly, the optimum reaction temperature of the protein is 37 °C, which matches the RPA reaction temperature. T4 gb32 also plays an important role in the chain transfer of T4 UvsX [53,54]. The Bsu DNA polymerase is a DNA isothermal amplification polymerase with strand replacement activity. The polymerase retains the 5′–3′ polymerase activity of Bsu I but lacks the 5′–3′ exonuclease domain, which can be used for recombinant enzyme amplification due to the 3′–5′ exonuclease activity. The interaction between T4 gb32 and the Bsu DNA polymerase can amplify a discrete product of thousands of bases with a pair of specific primers [55]. Because T4 phage is not readily available, recombinant enzymes from bacteria or fungus, such as SC-recA, BS-recA and Rad51, are now used, which are called RAA [56].

#### 2.3.2. Reaction Principle and Improved Methods 

**Figure 3:**
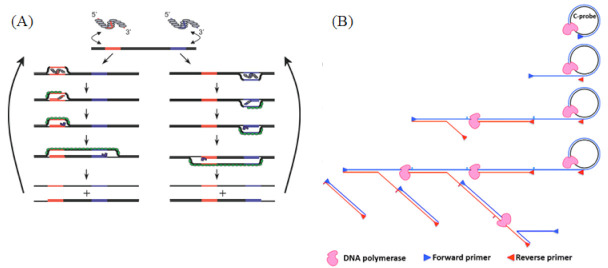
(**A**) RPA reaction mechanism. A recombinase enzyme binds to primers to form complexes that search for homologous sequences in the dsDNA target template and undergo strand displacement, in which single-stranded binding proteins (SSBs) bind to replaced DNA strands to prevent chain recombination, and Bsu polymerase amplifies the template. Reprinted with permission from Ref. [48]. Copyright 2006 Piepenburg et al. (**B**) Schematics of HRCA mechanisms. In vitro isothermal linear amplification of the circular DNA template was achieved by the chain displacement synthesis of a primer and circular DNA template under the action of a DNA polymerase with chain displacement activity. Reprinted with permission from Ref. [60]. Copyright 2014, Yan et al.

RPA is an isothermal amplification method that can be combined with fluorescent probes for real-time detection [48]. RPA’s reaction temperature is between 37 °C and 42 °C. In the presence of adenosine triphosphate (ATP), the recombinase combines with primers to form a protein–DNA complex. The complex then looks for the homologous sequence in dsDNA. Once the homologous sequence is determined by the primer, the complex will carry out strand exchange at the homologous site. The replaced chain is combined with SSB to prevent further replacement. Finally, the recombinase disintegrates, and the 3′terminal of the primer is replaced by the chain and binds to the Bsu DNA polymerase; finally, the primer is extended to synthesize DNA (Figure 3A).

The primers for RPA are relatively long, usually 30–38 bp. If the primers are not restricted, non-specific amplification can occur when detecting a large amount of background DNA. Luo et al. reported a new RPA method called betaine-assisted RPA (B-RPA) [57]. The addition of betaine in an RPA reaction significantly improves the specificity of RPA and reduces non-specific amplification. Specifically, the addition of 0.8 M betaine can significantly enhance the specificity and efficiency of RPA. In addition, when RPA is combined with a CRISPR-Cas system, it can improve the specificity and sensitivity. Feng et al. successfully integrated RT-RPA and CRISPR-Cas into a single test tube at one temperature (40 °C), which can specifically detect more than 200 S-gene sequences of SARS-CoV-2 within 5–30 min [58]. Xiong et al. reported a CRISPR/Cas9-mediated triple-line lateral flow assay (TL-LFA) combined with multiplex RT-RPA for the rapid and simultaneous dual-gene detection of SARS-CoV-2 in a single strip test [59]. However, without the thermal cycle of PCR to avoid binding between primers, it is difficult to avoid partial non-specific amplification at a constant temperature. In addition, agarose gel electrophoresis cannot be performed directly, and product purification is required before detection.

### 2.4. Rolling Circle Amplification

#### 2.4.1. Enzymes

Salas and colleagues developed RCA in 1989; it is one of the earliest IATs, based on the principle of rolling circle replication [61,62,63]. RCA was initially inspired by the natural rolling transcription of circular plasmids and virus genomes, producing an amplicon around cyclic DNA molecule templates, and this is now used in more and more diagnostic fields. The whole reaction is circular amplification, and the core enzyme used is the phi29 DNA polymerase. The phi29 polymerase is a small DNA-dependent polymerase that belongs to eukaryotic B-type DNA polymerases and is derived from Bacillus subtilis. The phi29 polymerase is often used in rolling circle amplification (RCA) due to its ability to produce linear single-stranded DNA amplifiers and to it having a reaction temperature of 35–40 °C. Furthermore, it has a remarkable replication processivity and nucleotide incorporation rate, and it is equipped with efficient strand displacement activity, which can be synthesized to exceed the length of 70 kb [61,64,65]. In addition, the phi29 DNA polymerase has a 3′–5′ exonuclease activity, which provides a high replication fidelity. When long-stranded gDNA are applied to ultrasonication, they break down into small DNA fragments that are then hybridized with the linear padlock probes to trigger the formation of circular RCA templates. The tails protruding from the 3′-end of the target DNA sequences are then digested by the 3′→5′ exonuclease activity of the phi29 DNA polymerase, even if they are double-stranded [62,66]. Lagunavicius used the phi29 DNA polymerase to transform target RNA into a primer, and the padlock probe can directly target the internal RNA sequence [67].

#### 2.4.2. Reaction Principle and Improved Methods 

RCA is divided into linear RCA and exponential RCA. In linear RCA, also known as single-primer RCA, the whole reaction is extended along the ring by a primer binding to the circular DNA template. The polymerase uses dNTPs to add nucleotides in the direction of 5′ to 3′, and the product is a tandem repeat copy that is thousands of times the length of the single ring (Figure 3B). The biggest advantage of linear RCA is that the signal is easily fixed, because the amplification product is always linked to the initial primer, so it is also very suitable for amplification detection on microarrays [68]. Exponential RCA is also known as hyperbranching RCA (HRCA). In HRCA, one primer amplifies the RCA product, and the second primer hybridizes with the RCA product and extends, replacing the extension strands of the reverse primers that had been bound to the RCA product. It is repeatedly extended and replaced to produce a dendritic RCA product and can be directly sequenced after phosphorylation (Figure 3B). However, RCA’s lock probe is usually close to 100 bp, creating a background interference in signal detection.

RCA has a high amplification efficiency and wide application but lacks a high sensitivity. Therefore, in order to improve the sensitivity of detection, Zhang et al. reported the use of stem-loop primers (SLP) to detect nucleic acids. The linear or exponential amplification (SLP-lRCA and SLP-eRCA) can detect the target DNA in liquid and solid phases [69]. This technique has been validated by detecting the presence of six different types of human papillomaviruses (HPVs). In addition, Zhang et al. reported a method of combining RCA and the CRISPR/Cas system, which achieved a high sensitivity of 34.7 fM due to the bi-specificity recognition of the RCA response initiated by the miRNA-padlock and specific cleavage of CRISPR-Cas12a [70]. Moreover, Lu proposed a fishhook probe-based RCA amplification technique (FP-RCA) that does not require total RNA extraction and can directly obtain targeted miRNAs from samples, reducing non-specific amplification [71].

### 2.5. Strand Displacement Amplification

#### 2.5.1. Enzymes

Strand displacement amplification, which was proposed by Walker in 1992, is one of the earliest isothermal amplification techniques. The SDA originally proposed an initial denaturation step at 95 °C, so at first, it was not considered to be a real isothermal amplification technique, but with the development of technology, additional “bumper” primers were added to the system to complete denaturation and achieve real isothermal amplification. The core enzymes of SDA reaction include the restriction endonuclease and a DNA polymerase with strand replacement activity. The restriction endonuclease is a bacterium that recognizes a specific base sequence in DNA and then cuts the DNA double strand. Moreover, restriction endonucleases cut only foreign DNA, not their own DNA molecules. Restriction enzymes used in cutting sires of restriction endonuclease need to be highly specific. The sensitivity to the hemiphosphorothioate recognition site can be rapidly dissociated to allow for DNA polymerase access after opening the gap. Many restriction endonucleases can effectively recognize the cutting site, such as HincII, a commonly used restriction enzyme in SDA that identifies restriction sites and cleaves DNA chains [72]. Exo-Klenow is a large segment of E. coli DNA polymerase I and is a DNA polymerase commonly used in SDA. Exo-Klenow lacks exonuclease activity due to the primer degradation from the 3′–5′ exonuclease activity during the reaction.

#### 2.5.2. Reaction Principle and Improved Methods 

**Figure 4:**
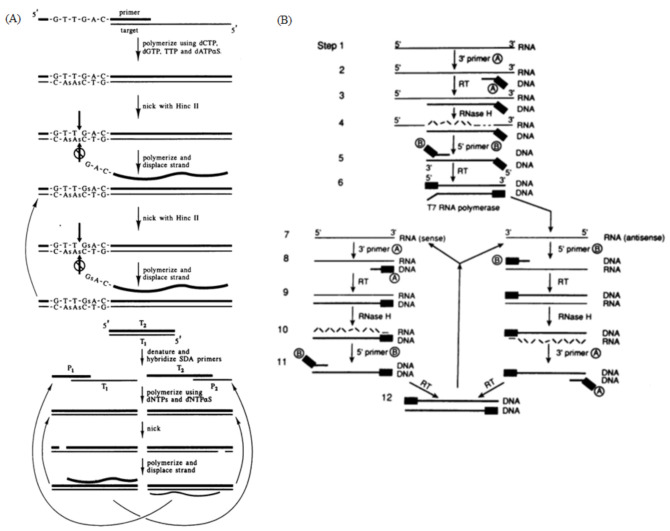
(**A**) Principle of SDA, generation of target amplification and exponential amplification. The two pairs of primers extend into double strands under the catalysis of the polymerase, and the endonuclease then cuts the newly synthesized strands between the primer and the template, allowing the new polymerase to bind and amplify the template while replacing the old strand. Reprinted with permission from Ref [50]. Copyright 1992, Walker et al. (**B**) Schematic of the NASBA mechanism. A reverse transcription primer containing the T7 promoter was used to reverse-transcribe the RNA target into a cDNA containing the T7 promoter, and then the cDNA was transcribed by the T7 RNA polymerase to obtain a large number of RNA amplification products; the synthesized RNA amplification products could re-enter the reverse transcription and transcription amplification cycle. Reprinted with permission from Ref. [51]. Copyright 1990, Guatelli et al.

SDA is an isothermal amplification method dependent on the DNA polymerase, with the whole reaction temperature being around 37 °C. The reaction process consists of three stages: the preparation of a single-stranded DNA template, the generation of a target DNA fragment with restriction sites at both ends and SDA. This method consists of two sets of primers: one located laterally (P1) and the second set, with 5′ restriction sites located medially (P2); P1 and P2 both contain endonuclease recognition sequences. At the beginning of the reaction, P1 and P2 are complementary to the template chain and extended into double chains under the catalysis of the polymerase. The endonuclease then recognizes the restriction sites at both ends of the double chain and cuts to form sticky ends. The second pair of primers bind to the end of the template chain, and a single chain is substituted by the polymerase to synthesize a new chain (Figure 4A) [72]. At present, SDA is extensively used in infectious disease detection.

Due to the uneven product fragment of SDA, a trailing phenomenon inevitably occurs during electrophoresis detection. In addition, SDA is too dependent on restriction enzymes, which can only cut at specific sites, and the two ends of the product carry the recognition sequence of restriction enzymes. Therefore, SDA products cannot be directly used for cloning and have no advantages in genetic engineering. Walker combined SDA with fluorescence polarization to detect the specific DNA sequence of Mycobacterium tuberculosis [73]. This method not only solves the problem of electrophoresis trailing but also allows for a more rapid and sensitive detection. Spargo reported an SDA reaction system in which BsoBI replaces HincII’s 5′–3′ exonuclease activity with exo-Bca instead of exo-Klenow [21]. This system can increase the reaction temperature to 60 °C and also shorten the reaction time to 15 min, thus reducing non-specific amplification and increasing the amplification efficiency by 100 times. Liu improved the efficiency of the hairpin probe and increased the sensitivity of the detection target, miR-21, by five times through altering the primers′ hybridization location, known as the “invading stacking primer” (IS-Primer)” [74]. Furthermore, Zhou proposed a fluorescence method for detecting miRNAs based on SDA and RCA, which has been successfully applied for detecting miRNA-21 in the serum of healthy and breast cancer patients, with a detection limit as low as 1.0 fM [75].

### 2.6. Nucleic Acid Sequence-Based Amplification

#### 2.6.1. Enzymes

Compton invented NASBA by the isothermal amplification of ssRNA in vitro in 1991 [23]. It mimics the replication of retrovirus RNA and produces ssRNA products, which has led to its popularity in the detection of single-stranded virus RNA or endogenous RNA, such as mRNA and miRNA detection. The enzymes that are critical to the reaction are Avian myeloblastosis virus (AMV) reverse transcriptase, ribonuclease H and the T7 RNA polymerase. AMV reverse transcriptase is the most commonly used enzyme in molecular biology. AMV is an alpha retrovirus that induces myeoloblastosis in chicken [76,77]. AMV RT consists of α and β subunits, with α subunits providing reverse transcriptase and RNaseH activity. RNaseH can catalyze RNA chains, synthesize cDNA chains and specifically degrade RNA residues in nucleic acids [78,79,80]. RNaseH can also remove both the RNA probe from the previous hybridization and the poly A tail from the mRNAs’ 3′ end [81]. The T7 RNA polymerase is the main product of the T7 bacteriophage. It is a DNA-dependent 5′–3′ RNA polymerase that specifically recognizes the sequence of the T7 promoter. It is a highly promoter-specific polymerase and only transcribes DNA downstream of the T7 promoter [82,83]. Moreover, it can recognize the modified NTP and can be used for the synthesis of various marker RNA. The T7 RNA polymerase has been widely used in industrial biotechnology, eukaryotic environment expression, synthetic gene circuits and RNA editing [84].

#### 2.6.2. Reaction Principle and Improved Methods

NASBA reaction is divided into two parts to detect the ssRNA virus: acyclic and cyclic (Figure 4B). In the reaction, the forward primer binds to the RNA chain, which is catalyzed by the AMV enzyme to form a DNA-RNA double chain. RNaseH digests the RNA in the double-strand hybridization while retaining the single strand of DNA. Under the action of reverse primers and the AMV enzyme, the DNA double-strand-containing T7 promoter sequence is formed. Foreign double-stranded DNA does not contain a T7 promoter sequence and cannot be amplified, thus giving NASBA a high specificity and sensitivity. The transcription process is completed under the action of the T7 RNA polymerase, with a large number of RNA targets produced. The amplified products of NASBA can be detected by agarose gel electrophoresis, electrochemiluminescence (ECL) and real-time fluorescence [85,86,87].

NASBA is mainly used for RNA detection; thus, RNA enzyme inhibitors are needed to prevent RNA degradation during the reaction. Moreover, an initial heating step is required before the reaction begins, prohibiting the amplification of dsDNA [88]. Real-time NASBA (QT-NASBA) has been successfully integrated into microfluidic systems. Dimov et al. used QT-NASBA to produce pathogen-specific responses from the chip-purified RNA of 100 lytic bacteria in less than 3 min [89]. Both techniques show a significant correlation with microscopic parasite counts, and the quantification results of the two real-time assays are significantly correlated for in vitro as well as in vivo samples. However, in comparison to QT-PCR, the results of QT-NASBA can be obtained 12 h earlier, with relatively easy RNA extraction and the use of finger prick blood samples [90]. NASBA also has inevitable disadvantages; its reaction components require three enzymes, which increases the cost, and, more importantly, it is not suitable for the detection of DNA viruses.

## 3. Enzymes-CRISPR

**Figure 5:**
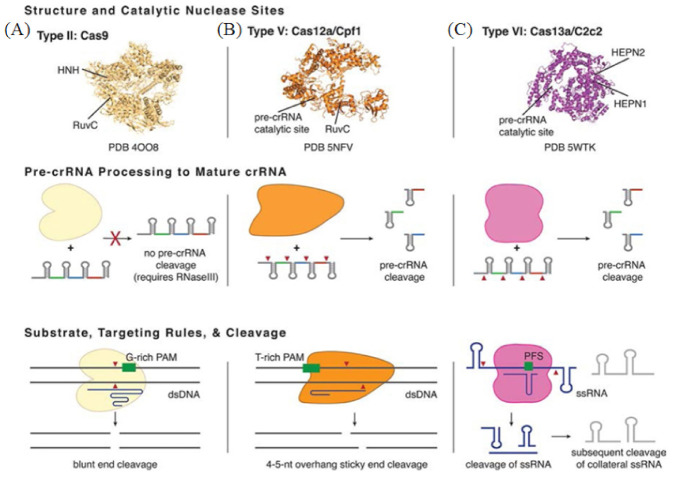
Basic structure and functions of Class 2 CRISPR-Cas Types. (**A**) The representative of Type II is Cas9 from Streptococcus pyogenes. (**B**) The representative of Type V is Cas12a from Francisella novicida U112. (**C**) The representative of Type VI is Cas13a from Leptotrichia shahii. Reprinted with permission from Ref. [96]. Copyright 2018, Pyzocha et al.

Clustered regularly interspaced short palindromic repeats (CRISPR) is a technology for editing genomes, allowing researchers to alter DNA sequences and modify gene function. The technology was adapted from a virus defense mechanism formed by bacteria and archaea in the process of biological evolution. Zinc-finger nucleases (ZFN) and transcription activator-like effector nucleases (TALEN) were the first genome editing nucleases to hit the market. CRISPR-Cas is the preferred gene-editing method currently, as it offers many advantages over ZFN and TALEN. These advantages consist of the target design simplicity due to it relying on the ribonucleotide complex rather than protein/DNA recognition, the higher efficiency due to it eliminating the need for transfection and, finally, the ability to multiplex genes together. The CRISPR-Cas system first integrates the foreign nucleic acid fragments into the palindromic repeat sequence. When these foreign nucleic acids invade the body again, the CRISPR RNA (crRNA) expressed by the cell binds to the Cas protein and quickly cleaves the foreign nucleic acid [91,92]. CRISPR-Cas systems are categorized into two classes: Class 1 and Class 2, which are further subdivided into types and subtypes [93]. Class 1 is more common in bacteria and archaea, but its composition is more complex and less studied [94]. Class 2 is more widely known due to it requiring only one Cas protein to cut the target sequence. Class 2 is a commonly used gene editing tool which has become a hot spot in molecular diagnostics (Figure 5) [95]. Below, we will focus on four different Cas effector proteins: Cas9, Cas12, Cas13 and Cas14.

### 3.1. Cas9

**Figure 6:**
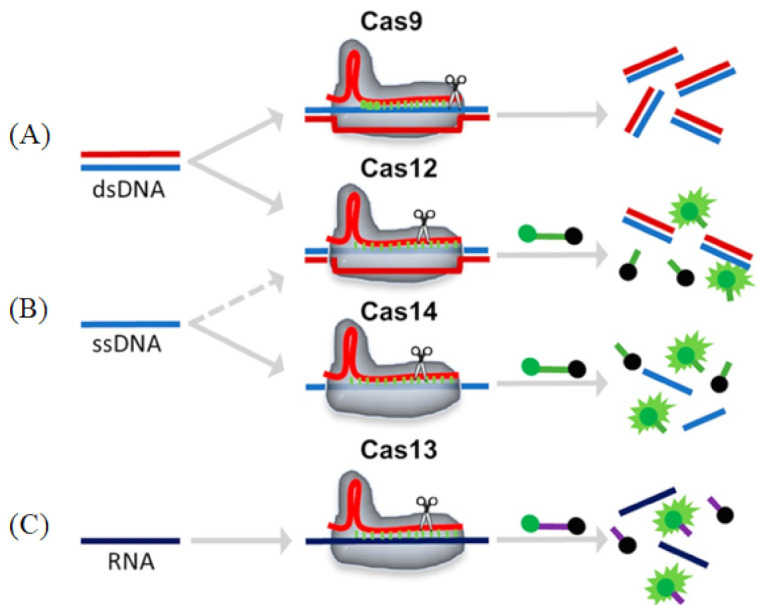
Schematic representations of different Cas enzyme activities. Reprinted with permission from Ref. [99]. Copyright 2020, Suea-Ngam et al. (**A**) Cas9: The target dsDNA is cis-cleavage after binding to the gRNA-Cas9 complex. (**B**) Cas12/14: The target ds/ssDNA binds to the gRNA-Cas12 complex and is cis-cleavage, followed by an ssDNA that is trans-cleavage and releases a fluorescence signal. (**C**) Cas13: The target RNA binds to the gRNA-Cas13 complex and is cis-cleavage, followed by an ssRNA that is trans-cleavage and releases a fluorescence signal.

Cas9 (class 2, type II) is an RNA-mediated endonuclease, which is primarily used to cleave dsDNA and is thought to be the sole protein responsible for CRISPR RNA (crRNA)-guided silencing of foreign DNA. DNA recognition by Cas9 enzymes requires a protospacer adjacent motif (PAM) next to the target site. With the use of the RNA-guided PAM, Cas9 is able to cleave both ssDNA and ssRNA. crRNA and trans-activating crRNA (tracrRNA) form a two-RNA structure that guides Cas9 to introduce double-stranded breaks in target DNA [97]. Importantly, the connection between crRNA and tracrRNA may have been artificially modified to form a single guide RNA(gRNA) [98]. Cas9 can be cut at a specific position of the target DNA under the guidance of gRNA (Figure 6A). Cas9 has HNH and RuvC cutting domains, which cut both the complementary and non-complementary DNA strands.

### 3.2. Cas12

Cas12 is a large diagnostic protein in the CRISPR system. Here, we introduce the commonly used Cas12a, previously known as Cpf1. Cas12a (class 2, type V) is an RNA-mediated DNA endonuclease similar to Cas9. Cas12a cleaves pre-crRNA and generates intermediate crRNAs that are processed further, leading to mature crRNAs. Cas12a initiates DNA cutting by forming an R-loop where gRNA displaces one strand of a DNA substrate, positioning the site for first-strand cleavage (Figure 6B). Compared to Cas9, Cas12a only needs the crRNA and does not need the tracrRNA, and it is also involved in the maturation process of pre-crRNA. Another advantage to Cas9 is that Cas12a is smaller and is thus more easily transferred to cells [100,101]. Cas12a contains the RuvC domain but lacks a detectable second endonuclease domain, whereas Cas9 uses the HNH and RuvC endonuclease domains to cleave the target and non-target DNA strands, respectively. Together, these observations imply major differences in the target DNA recognition and cleavage mechanisms between Cas9 and Cas12a [98,101,102]. In addition, the cleavage site of Cas12a is far from its recognition site, so it can be edited many times in succession [103].

### 3.3. Cas13

Cas13 (class 2, type VI) is dependent on RNA-mediated RNA endonuclease, which is used to specifically target ssRNA (Figure 6C). Cas13, formerly known as C2c2, is a single protein composed of multiple domains, recognizing crRNA and cleaving both RNA and pre-crRNA [104,105]. The cleavage activity of Cas13 depends on two higher eukaryotes and prokaryotes nucleotide-binding (HEPN) domains. These domains form a ribonuclease active site, allowing it to act as an RNA-guided RNA endonuclease. The type VI family also includes CRISPR arrays. A CRISPR array is a repeating genomic sequence that contains multiple copies of a short segment of DNA alternating between repeat and spacer sequences. CRISPR arrays transcribe to form pre-crRNA, which is then processed by Cas proteins to form mature crRNA. Cas13 binds to the crRNA to form a binary complex. The crRNA is responsible for the specific recognition of the substrate. Once recognition occurs, it will form a Cas13-crRNA-target ternary complex, and Cas13 can be activated to cleave the substrate. Cas13a has been used to detect viruses, virus resistance mutations and clinically relevant virus single-nucleotide polymorphisms (SNPs) in body fluids.

### 3.4. Cas14

Cas14 (class 2, type V) is the smallest diagnostic protein, with a range of 400–700 amino acids in length, half the size of previous class 2 proteins [106]. There are 24 different gene variants in Cas14, which are divided into three subgroups: Cas14a, Cas14b and Cas14c. All of these variants have the RuvC nuclease domain, which is characteristic of the CRISPR-Cas enzyme. However, unlike other Cas enzymes, Cas14 does not exist in the bacterial genome, only in the archaea genome [107]. Interestingly, and unlike other class 2 proteins, researchers could not detect a PAM binding requirement for Cas14. Cas14 can only cleave ssDNA and not dsDNA nor ssRNA (Figure 6C). Cas14 is also highly susceptible to mismatches. Therefore, Cas14 has the advantage of sequence detection, specifically distinguishing SNPs (Cas14-DETECTR) [106]. Cas14-DETECTR can be used for the rapid and simple real-time detection of a small amount of DNA in clinical samples, and it is suitable for the diagnosis of cancer and infectious diseases.

Although CRISPR has been used in labs around the world for scientific research, its drawbacks are clear. CRISPR is not sensitive enough on its own and needs to be combined with other amplification techniques to complete the whole amplification reaction, such as RPA and LAMP. For example, Zhang et al. proposed using CRISPR-LAMP to detect the SARS-CoV-2 Delta and Omicron variants [108]. Qin et al. reported on the detection of African swine fever virus using CRISPR-RPA [109]. In addition, the dependence of the PAM sequence also limits its application, which can cause the non-specific cutting and mutation of non-targeted sites in the genome, resulting in an off-target phenomenon and affecting the editing efficiency. Therefore, increasing the specificity and reducing the off-target effect is a critical step for future CRISPR applications.

## 4. Advances in Clinical Application

Thanks to the simple operation and high amplification efficiency, NAATs have become a major component in clinical diagnosis. With the developments in molecular biology and the continuous optimization of nuclease enzymes and reaction conditions, the advancement of rapid diagnostic methods is continuously growing, especially in infectious diseases, tumors, chronic diseases and individualized treatment. In this section, we will summarize the application of NAATs in clinical diagnosis.

### 4.1. Infectious Disease

Infectious diseases are still one of the biggest threats to human health and safety. Improving the rapid detection of pathogens has a significant effect on the treatment of infectious diseases [110,111]. Traditional NAATs have a lower efficiency and specificity, which means they struggle to meet the needs of the clinical market. Newer NAATs such as isothermal amplification can detect microbial pathogens causing infectious diseases and can quantitatively detect the reproduction or replication of pathogens in the body [2].

The highly contagious and pathogenic 2019 coronavirus, known as Severe Acute Respiratory Syndrome Coronavirus 2 (SARS-CoV-2), which began to appear in Wuhan, China at the end of 2019 and has since spread worldwide, is constantly mutating [112,113,114,115,116]. Therefore, rapid and highly sensitive detection is of the upmost importance. The global outbreak has dramatically increased the number of cases; thus, RT-PCR is not suitable for on-site diagnosis. Therefore, rapid and simple amplification detection techniques are in high demand. Lau et al. proposed a rapid and sensitive RT-LAMP method for detecting SARS-CoV-2, which detected one copy of SARS-CoV-2 RNA in 30 min [117]. Lu et al. proposed a sensitive RT-LAMP detection method with a limit of detection (LOD) of 118.6 copies of SARS-CoV-2 per 25 ul reaction [118]. Shelite et al. developed the first isothermal amplification test based on RPA and side stream pairing, which can be used for the rapid detection of SARS-CoV-2 with an LOD of 35.4 viral cDNA nucleocapsid (N) gene copies/μL [119]. Zhang’s team combined Cas13a with RPA to establish a diagnostic method based on CRISPR, termed Specific High-Sensitivity Enzymatic Reporter Unlocking (SHERLOCK). SHERLOCK can successfully detect specific strains of the Zika virus and Dengue virus, distinguish pathogenic bacteria and human genotype DNA and identify mutations in acellular tumor DNA [120]. Zhang used an improved SHERLOCK to detect SARS-CoV-2 within an hour [121]. In addition, Patchsung et al. also reported using the SHERLOCK assay to detect SARS-CoV-2 using the enzyme Cas13a, with a comparable result to that of Qpcr [122]. These detection methods are suitable for on-site diagnosis and areas where resources are scarce. Magro et al. used reverse transcriptase polymerase amplification (RT-RPA) and a paper microfluidic device to amplify and detect the synthetic RNA of the Ebola virus within minutes [123]. Zhou et al. developed an amplification technology based on real-time nucleic acid sequence-based amplification (RT-NASBA) for the rapid detection of Japanese encephalitis virus (JEV), with an LOD of six copies per reaction [124]. Zhao and co-workers developed a lateral-flow biosensor based on magnetic beads and strand displacement amplification to detect hemorrhagic fever virus, with an LOD of 10 fM [125]. Doudna’s team combined Cas12a with isothermal amplification, known as DNA endonuclease-targeted CRISPR trans reporter (DETECTR), to establish a diagnostic method for rapid and specific detection [126].

The most valuable aspect of PCR in medical testing is the diagnosis of infectious diseases, such as hepatitis B virus (HBV). Huang found that the HBV copy number was positively correlated with the liver cancer grade and tumor lymph node metastasis using ddPCR [127]. Interestingly, ddPCR only needs 0.54–0.594 copies of covalently closed circular DNA (cccDNA) to detect HBV and can directly quantify nucleic acids at the single molecular level [127,128]. Furthermore, Zhang and colleagues developed a lateral flow test strip combined with a recombinant polymerase amplification assay for the rapid detection of HBV. This method takes about 70 min from nucleic acid extraction to endpoint detection, can detect as few as 10 copies/reaction of HBV and has no cross reaction with other common pathogens [129]. Pathogen quantifications are correlated with the severity, infectivity and treatment effect of infectious diseases. In recent years, NAAT has continuously improved and innovated its specificity, sensitivity and repeatability; therefore, its application in the field of clinical diagnosis has become more and more extensive.

### 4.2. Tumor Diagnosis

Over time, cancer cells develop from a mutated cell to a visible tumor. Improving the LOD and early diagnosis of cancer is helpful for the choice of treatment and can also improve the survival rate of cancer patients. Pathogens are inextricably linked to human health, such as Helicobacter pylori and gastric cancer [130,131,132]. RCA is used for the electrochemical detection of circulating tumor cells (CTCs). Shelite et al. showed that, when combining RCA, magnetic nanospheres and the electrochemical current generated by DNA, efficient magnetic capture and the hypersensitive detection of CTCs can be achieved [133]. Lv et al. used a rapid and sensitive CRISPR-Cas12a system to detect CTCs, with an LOD of 26 cells mL^−1^. This method can also directly detect CTCs in human blood, which has great potential in liquid biopsy [134]. MicroRNAs (miRNAs) are short non-coding RNAs, which play an important role in the regulation of gene expression, cell development, differentiation and function [135,136,137]. The dysfunction of miRNAs can also interfere with the expression of carcinogenic or tumor suppressor target genes [138,139,140]. Therefore, miRNAs are considered to be an important biomarker in clinical applications. Tian et al. combined RCA with LAMP to establish a rapid and sensitive method for detecting miRNAs [141]. Zhao et al. proposed a microfluidic surface-enhanced Raman scattering (SERS) sensor based on RCA and a tyramine signal amplification (TSA) strategy for detecting exosomal miRNA. The SERS significantly improved the sensitivity of exosomal miRNA analysis, and the detection limit was as low as 1 pmol/L. It was successfully applied to the analysis of exocrine secreted by breast tumor cells [142]. Mader et al. proposed an NASBA detection method for the simultaneous expansion of miRNAs and mRNA sequences in 2012 [143]. Wang et al. also successfully reported an RCA-assisted CRISPR-Cas9 method for detecting miRNAs [144]. In addition to using Cas9 and Cas12a, Cas13 is also commonly used in the detection of miRNAs. Cui proposed a method of combining the CRISPR-Cas13 system with catalytic hairpin assembly (CHA) to detect miRNAs. This method has a high sensitivity, with an LOD of 2.6 fM, and thus has great applications in early clinical diagnosis [145]. Chen et al. proposed a label-free and enzyme-free fluorescence strategy based on SDA, using sulfhydryl functionalized CD (CDs-SH) as a probe for the highly sensitive detection of miRNA, with an LOD of 0.03 pM [146]. Moreover, the detection and imaging of miRNA in living cancer cells by a decomposing plasma core satellite probe and SDA have also been proposed, with an miRNA LOD of 2 pM [147]. Chang et al. reported a novel photoelectrochemical (PEC) miRNAs biosensor based on SDA reaction for analyzing miRNA-21 and let-7a in breast cancer. This PEC biosensor can detect miRNA-21 and let-7a simultaneously with a high sensitivity and detection limits of 6.6 fmol/L and 15.4 fmol/L based on 3σ [148]. Wang et al. proposed a hypersensitive miRNA detection method combining asymmetric polymerase chain reaction (A-PCR) and LAMP. The detection can be completed in 90 min, and the detection limit is as low as 10 amol/L [149]. All these methods improve the ability of achieving early cancer diagnostics.

In addition to the early diagnosis of a tumor, the expression of drug-resistant genes in tumor chemotherapy is also an important issue. Claudia used RT-PCR and qRT-PCR to detect drug pump expression and found that the combination of low-dose conventional chemotherapy and an M2 dual steric agonist may be a new pharmacological approach to reducing the Glioblastoma Stem Cells (GSCs) resistance in Glioblastoma multiforme (GBM) therapy [150]. PCR allows for the faster detection of tuberculosis (TB) and drug susceptibility testing (DST) for key drugs such as rifampin (RIF) and isoniazid (INH), allowing doctors to diagnose faster and offer a more effective treatment [151,152,153]. In addition, Gliddon developed a targeted RPA-nanopore sequencing workflow for the rapid prediction of the drug resistance of TB isolates [154]. Early detection and early treatment are the keys to patient survival.

### 4.3. Human Genetic Diseases

NAAT can also be used for the diagnosis of human genetic diseases. The most effective way to prevent the occurrence of genetic diseases is prenatal diagnosis and pre-symptomatic diagnosis. NAAT has a higher sensitivity and is faster than traditional methods of disease diagnosis [155,156]. For example, traditional prenatal diagnoses include amniocentesis, villus extraction, amniotic fluid or cord blood, all of which carry a risk of miscarriage. NAAT allows for non-invasive prenatal diagnosis to determine chromosomal abnormalities. Tan et al. reported a method based on multiplex ddPCR that can detect fetal aneuploidy before birth. This method can not only simplify the detection process but also improve the reusability and practicability [157]. Almasi et al. proposed an SRY gene to identify the sex determination of embryos in pregnant women at 8 weeks of pregnancy by LAMP [158]. Prenatal diagnosis has made a significant contribution to prioritizing prenatal care, and prenatal screening has become an important choice for most pregnant women.

The gene mapping of human genetic diseases is based on genealogical linkage analysis, but linkage analysis has been unable to meet all research needs. SNP is the simplest form of DNA variation among individuals. It is widely distributed in the human genome, and a large part of it has a direct impact on human diseases [159,160]. A genotyping method of SNPs for the rapid detection of multiple sample types is proposed [161]. Dhar et al. used CRISPR-Cas for the detection of SNPs [162]. In addition, Chen et al. proposed a CRISPR-Cas12a high-fidelity microfluidic chip detection of SNPs, which completes in 20 min, and the typing results are consistent with the sequencing results [163]. Ding et al. proposed a novel SNP typing method based on probe-enhanced LAMP (PE-LAMP) [164].

NAAT has many advantages in cases of highly genetic diseases or age-related diseases with a genetic predisposition. Xue reported a Taqman-MGB probe qPCR method that can be used for the qualitative and quantitative analysis of three major Leber hereditary optic neuropathy (LHON) mitochondrial DNA (mtDNA) mutations, providing a promising method for the genetic screening and detection of LHON mutations [165]. Sequence changes of Duchenne muscular dystrophy were detected by qPCR and sequencing, allowing for an early treatment and a better prognosis [166]. Chen et al. proposed nested PCR and multiplex ligation-dependent probe amplification (MLPA) for accurately diagnosing the genotype of HKαα carriers in patients with thalassemia [167]. The early screening of human genetic diseases plays an important role in preventing diseases and in the prognosis of the disease.

### 4.4. Chronic Disease

Elderly individuals are more prone to chronic diseases; thus, a global trend of researching the relationship between age and chronic diseases has become a hot topic. Chronic diseases are long-term, almost incurable non-communicable diseases affected by genetic, physiological, environmental and behavioral factors. These include cardiovascular and cerebrovascular diseases, diabetes, mental illness, rheumatoid arthritis and so on [168,169]. The proprotein convertase subtilisin/kexin type 9 (PCSK9) gene can bind to the low-density lipoproteins receptor (LDLR) domain, resulting in an increase in the level of circulating LDL-C and a higher level of LDL-C, allowing for an increased incidence of cardiovascular disease [170]. Musunuru et al. used CRISPR technology and lipid nanoparticles to accurately and effectively reduce the level of PCSK9 in the liver by about 90%, and it remained stable within 8 months after a single dose of treatment [171]. The ratio of plasma Aβ42 to Aβ40 can be used as an accurate index for the diagnosis of Alzheimer’s disease. Wang et al. established a chemiluminescence immunoassay based on RCA (RCA-CLIA) for quantifying Aβ in plasma. The sensitivity of this method is higher than that of the traditional CLIA method, and there is no loss of specificity [172]. Chlamydia pneumoniae is closely related to coronary heart disease. Coombes et al. was able to detect C. pneumoniae ompA mRNA transcripts with NASBA and an aequorin bioluminescent hybridization assay. This method has an LOD of 0.2 IFU, and its sensitivity is at least 10 times higher than that of Northern blot detection [173].

### 4.5. Personalized Medicine

Individualized treatment is based on each patient’s disease information to determine the treatment policy so as to carry out the most appropriate drug therapy. Targeted medicine is the main application of NAAT in individualized diagnosis and therapy. At present, there are many tumor molecular targets in clinical targeted therapy, including EGFR, KRAS, HER-2 and more [174,175]. Xue reported a Taqman-MGB nanoPCR system for detecting EGFR and improving the specificity for single-base mutation detection. They demonstrated an improvement in specificity across a wide concentration range from 10−9 μM to 10 μM and detected a mutation abundance as low as 0.95% in spiked samples, which is lower than that of existing PCR methods [176]. RT-PCR and Sanger sequencing were used to detect KRAS mutations. This is useful in identifying patients with a poor prognosis for further interventions [177]. In addition, multiplex picodroplet digital PCR was also used to detect KRAS [178]. Human copper transporter 1 (hCTR1) was detected by RT-LAMP within 45 min, which can be used for the diagnosis of cisplatin sensitivity in cervical cancer [179]. With the ability to detect multiple target systems for tumors, doctors can tailor treatment plans for each patient, improving efficacy and patient survival.

## 5. Conclusions

The development of molecular biology technology has provided a powerful tool for researchers in exploring the unknown aspects of diseases. The continuous development in science and technology has allowed newer and more improved NAATs to emerge. Even though PCR is the gold standard in the industry, due to its equipment requirements and high cost, it is not suitable in resource-poor areas. Isothermal amplification has the advantage of a lower cost and a more user-friendly interface. Furthermore, it does not require equipment dependence and is therefore well suited for field testing. In this review, we summarized the amplification technologies of infectious microorganisms, tumor etiology and diagnosis, human genetic disease diagnosis and the application of personalized therapeutics. The core component of these amplification technologies is the enzyme, and it is because of the clever combination of these enzymes that the various NAATs are achieved.

Although NAAT is a widely used method in clinical applications and fundamental research, it also has limitations such as false-positives and false-negatives. The future development direction of NAATs is simplification, automation and multiplex target amplification. As a new type of reaction and detection carrier, microfluidic chips can be combined with NAATs to develop a small and portable real-time detection platform. Moreover, microfluidic chips can also integrate multiple reactions to realize the simultaneous detection of multiple targets. In addition to the realization of multiplex target amplification under the condition of a single closed tube, a high sensitivity and specificity should also be achieved, which is also an urgent technical problem to be solved. It has been mentioned above that enzymes are the core of NAATs, so the performance improvement or optimization of them is also a big difficulty for future NAATs. The improved performance of the enzymes can also greatly improve the efficiency of NAATs and better meet the growing needs of healthcare diagnosis. With the continuous development of science, NAATs will gradually become the main means of clinical diagnosis, with their incomparable strong advantages.

## Figures and Tables

**Table 1:** Nucleic Acid Amplification Methods.

Nucleic Acid Amplification	Enzymes	Reaction Temperature (°C)	Reaction Time (min)	Target	Primers	Amplification Capacity	Refs.
PCR	Taq polymerase	95,55,72	45–120	DNA	2	107–1010	[7,14]
LAMP	Bst DNA Polymerase	60–65	60	DNA	4	109	[15,16]
RPA	Bsu DNA polymerase recombinase and ssDNA binding protein	37–42	20	DNA/RNA	2	10	[17,18]
RCA	Phi29 DNA Polymerase	30–65	60–180	DNA/RNA	2	109	[19,20]
SDA	Restriction endonuclease and DNA polymerase	37–40	120	DNA	4	107	[21,22]
NASBA	Reverse transcriptase and RNA polymerase (RNaseH)	41	60–120	RNA	2	106	[23,24]

## Data Availability

Not applicable.
